# Reproductive capacity and recurrence of disease after surgery for moderate and severe endometriosis – a retrospective single center analysis

**DOI:** 10.1186/s12905-020-01016-3

**Published:** 2020-07-13

**Authors:** Cordula Schippert, Yvonne Witte, Janina Bartels, Guillermo-José Garcia-Rocha, Matthias Jentschke, Peter Hillemanns, Sudip Kundu

**Affiliations:** Department of Obstetrics and Gynaecology, Hanover Medical School, Carl-Neuberg-Straße 1, 30625 Hannover, Germany

**Keywords:** Moderate endometriosis, Severe endometriosis, Recurrence, Pregnancy rate, Sterility, Reproduction, Surgical access

## Abstract

**Background:**

Endometriosis can be associated with considerable pain and sterility. After surgical excision of moderate or severe endometriosis lesions, the rate of recurrence reaches up to 67%. The objective of this retrospective study was to establish the recurrence and pregnancy rates following surgical resection of stage III/IV endometriosis lesions. Indications for operation were endometriosis symptoms, sonographic findings and/or infertility.

**Methods:**

A total of 456 patients who underwent stage III/IV endometriosis surgery between 2004 and 2014 were sent a questionnaire relating to their postoperative medical treatment, pregnancies, relief of symptoms and recurrence. Responses of 206 patients (45.2%) and their clinical data were analysed for this study.

**Results:**

A total of 66.5% (*N* = 137) of patients had stage III disease, and 33.5% (*N* = 69) had stage IV disease. The average age was 37 years (17–59). A total of 63.1% (*N* = 130) of surgeries were performed by laparoscopy, 21.8% (*N* = 45) were performed by laparotomy and 15% (*N* = 31) were performed by conversion. Complete resection of endometriosis lesions was achieved in 90.8% of patients (*N* = 187). After surgery, 48.5% (*N* = 100) of the women did not receive hormonal treatment; the main reason was the desire for children in 53%.

Complete or partial relief in complaints was achieved in 93.2% (*N* = 192). The rate of recurrence was 21.8% (*N* = 45). The statistically significant factors that was associated with a higher risk to develop recurrence was an age < 35 (*p* < 0.005).

After surgery, 65.8% (79/120) of patients who wished to have children became pregnant. There was a statistically significant association among a higher postoperative pregnancy rate and age < 35 (*p* < 0.003) in multivariate logistic regression analysis and laparoscopic surgical access in univariate logistic regression analysis (*p* < 0.01).

**Conclusion:**

We assessed the high percentage of complete or partial relief of symptoms of 93.2%, the high postoperative pregnancy rate of 65.8% and the low rate of recurrence of 21.8% compared to international literature to be very encouraging for women suffering from moderate and severe endometriosis. Though laparoscopy is considered the ‘gold standard’of endometriosis surgery, laparotomy still may be indicated in patients with extensive endometriosis especially to preserve reproductive function.

## Background

Endometriosis can significantly decrease the quality of life of the affected women [1]. The effect of endometriosis on the quality of life concerns not only severe pain during menstruation, urination, defecation and intercourse but also psychological and social status as well as family planning [2]. From the onset of the first symptoms until the correct diagnosis is established, up to 7 years pass by on average [3]. Often, the pain has already become chronic at the time patients with endometriosis are diagnosed and receive treatment [1].

The prevalence of endometriosis is estimated at a minimum of 5–15% of all women of reproductive age [4]. Endometriosis is established in up to 80% of women suffering from lower abdominal pain and dysmenorrhoea [4–6].. The rASRM classification on the staging of endometriosis is practicable to use and makes it easier for patients to understand the extent of their disease [6, 7]. However, retroperitoneal structures and deeply infiltrating endometriosis lesions are not considered [7], and there is only a remote association between rASRM stage and pain intensity and frequency [8].

### Endometriosis and infertility

Up to 50% of endometriosis patients suffer from infertility; in turn, up to 50% of the sterile women are diagnosed with endometriosis [6]. While the probability of achieving a pregnancy per menstruation cycle in healthy couples of reproductive age is between 15 and 20%, this is, by contrast, in the range of 2 and 10% for women with endometriosis [6, 9]. The exact causes for this decrease in fertility are not known. Peritubal and ovarian adhesions can affect the release and transport of the ovum. Increased inflammatory and angiogenic cytokines in the peritoneal fluid as well as autoantibodies to endometrial fragments may decrease fertility capacity [10]. Moreover, the ovarian reserve can be reduced in endometriosis patients. Studies show that patients with moderate or severe endometriosis without previous surgeries have lower levels of Anti-Mullerian Hormone (AMH) than healthy women [11, 12].

**Fig. 1:**
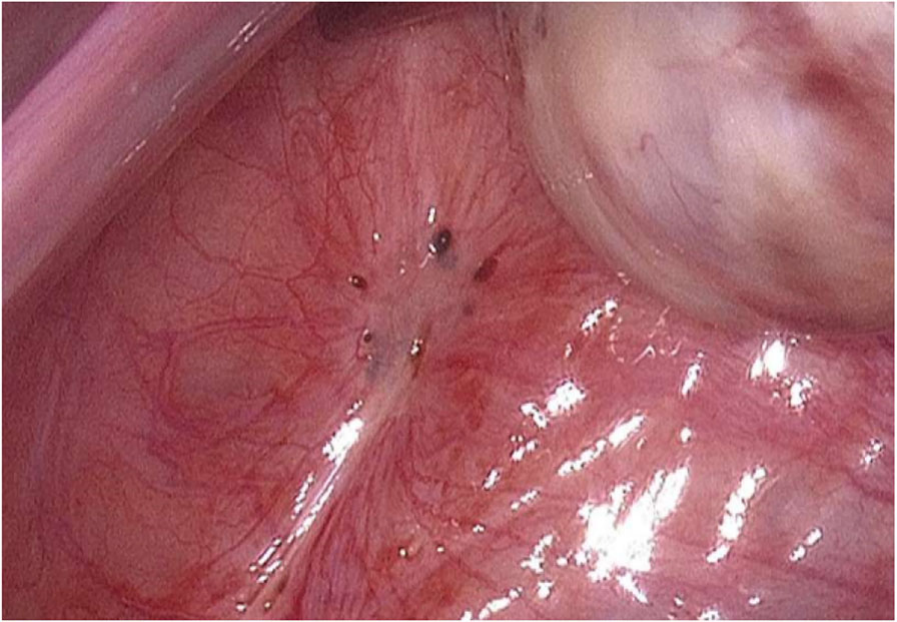
Peritoneal endometriosis of the left pelvic wall (copyright remains with the authors). Typical livid lesions and an increased vascular pattern are visible

The surgical removal of endometriosis lesions, which can help reduce pain and improve fertility, is globally recognized as first-line therapy. In peritoneal endometriosis (Fig. 1), the primary therapy objective is the removal of all visible lesions [13].

In ovarian endometrioma, the cyst wall should be surgically removed completely [14]. However, it should be kept in mind that the resection of ovarian endometrial cysts can lead to a diminished ovarian reserve and therefore to a decrease in fertility [12, 15]. ESHRE guidelines recommend laparoscopic resection of lesions of the disease to improve the spontaneous pregnancy rate in women with infertility with stage III or IV endometriosis [16]. In special cases of severe endometriosis with pronounced adhesions, tubal pathology or even recurrence, a laparotomy can also be indicated [17].

**Fig. 2:**
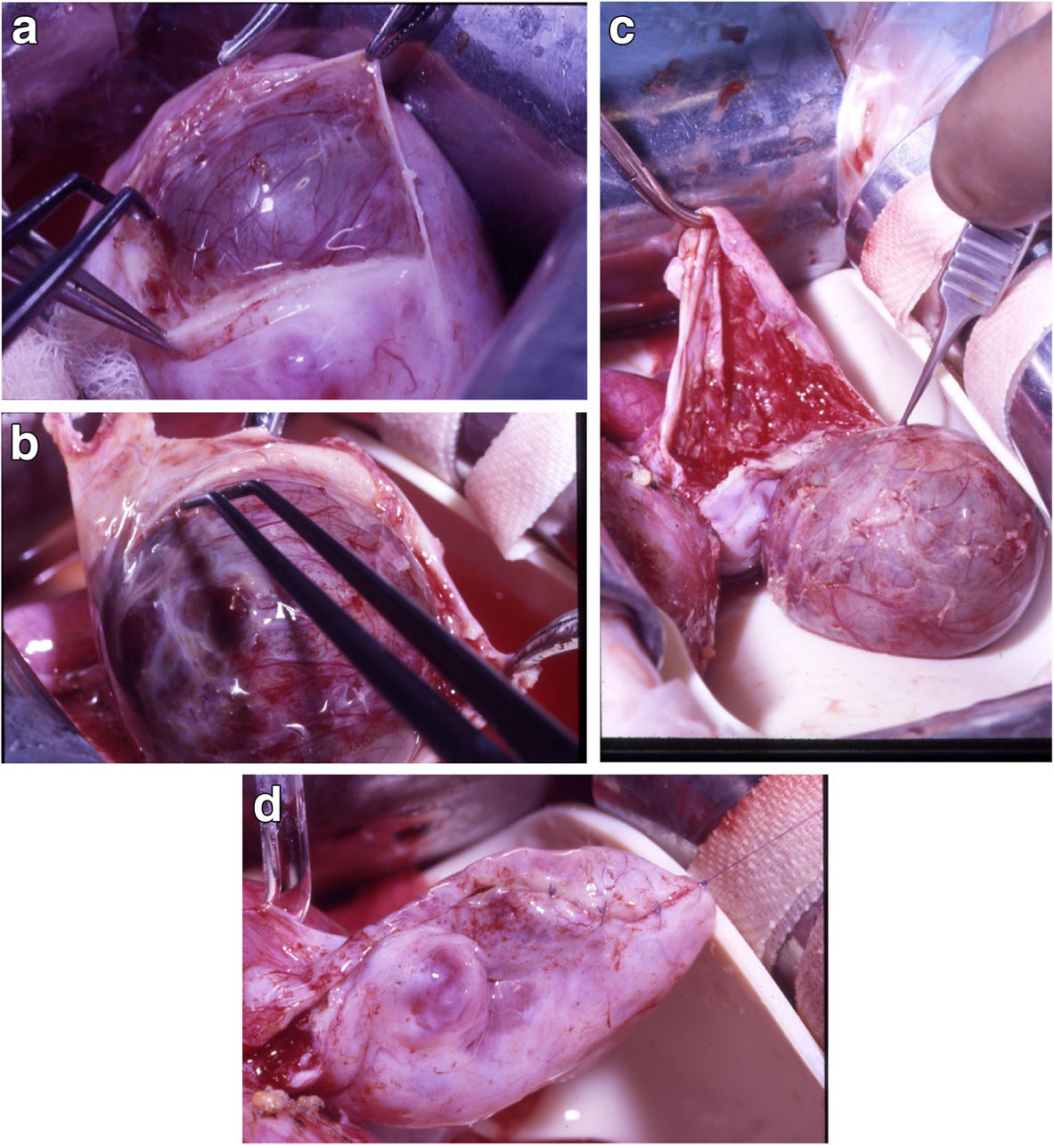
**a**: Opening of the ovary with underlying endometrial cyst (copyright remains with the authors). **b**: Careful preparation and subsequent enucleation of the cyst without rupture (copyright remains with the authors). **c**: Careful preparation and subsequent enucleation of the cyst without rupture (copyright remains with the authors). **d**: Reconstruction of the ovary with 8–0 Vicryl suture (copyright remains with the authors)

**Fig. 3:**
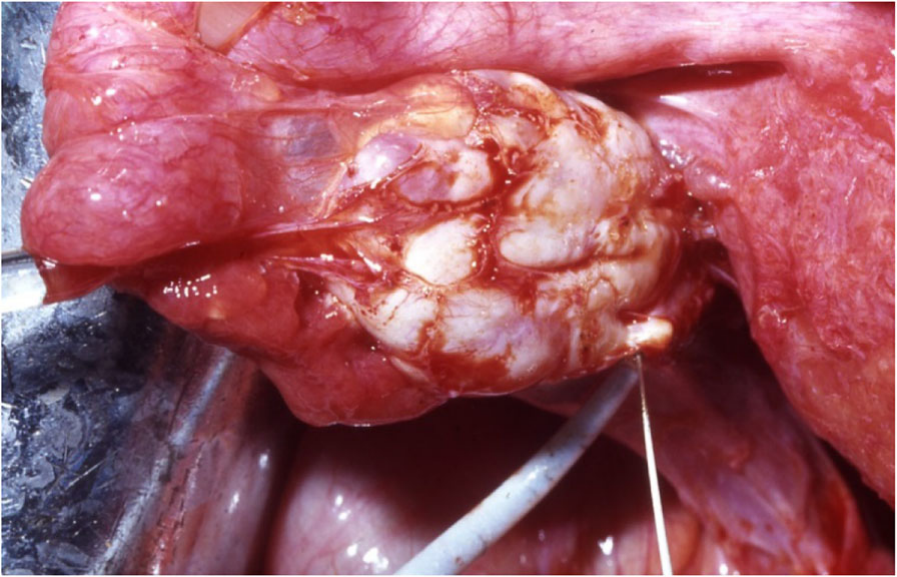
Firm, endometriosis-related, tubo-ovarian adhesions (copyright remains with the authors)

Ovarian endometriomas can be removed completely from the ovarian tissue without greater loss, as shown in the following Fig. 2a-d from our own hospital:

Extensive tubo-ovarian adhesions can be gently detached and the function of the fimbrial funnel can be restored so that spontaneous conception becomes possible (Fig. 3**).**

### Postoperative recurrence

Unfortunately, the rate of recurrence after endometriosis surgery is considerable at times and varies between 6 and 67% after 5 years [18–22]. The rate of recurrence increases in relation to the extent of endometriosis at baseline [18]. Preoperative hormone therapy leads to a significant decrease in the spread of the disease measured with the rASRM score, but does not affect the postoperative symptoms and pregnancy rates whereas postoperative pharmacological therapy significantly decreases the rate of recurrence [23].

### Study objective

The objective of this retrospective questionnaire-based study was to establish postoperative recurrence and pregnancy rates in patients after surgical resection of moderate and severe endometriosis lesions (rASRM stage III and IV). This was performed with a data analysis of patients who underwent surgery between 2004 and 2014 at the hospital. Indications for operation were typical endometriosis symptoms, sonographic findings (e.g., ovarian cysts) and/or infertility.

A subanalysis was used to study the extent of recurrence and pregnancy rates depending on disease stage and surgical access (laparotomy, conversion from laparoscopy to laparotomy), how often the surgery had to be extended by a bladder/intestinal surgical procedure and how often intra- and postoperative complications occurred.

## Methods

This study was a retrospective review conducted at the Department of Gynecology and Obstetrics at Hannover Medical School (MHH) and was approved by the Ethics Committee of the MHH (no. 3527–2017). Written informed consent to participate in the study was obtained from all participants.

All premenopausal women who underwent surgery for moderate or severe endometriosis (rASRM stage III and IV) within a period of 11 years (2004 until 2014) were included in the study. Premenopausal status was defined as regular periods, the non-necessity of hormone replacement therapy, and the absence of menopause symptoms such as hot flashes or mood swings. Women with common benign comorbidities such as hypertension, autoimmune diseases or thyroid function disorders were included, whereas patients with malignant disease were excluded from the study. The surgeries were not performed during menstruation; otherwise, they were performed independently from the phase of the menstrual cycle.

By means of the hospital internal documentation programmes, a high number of 456 patients were identified who underwent surgery for histologically established grade III or IV endometriosis and fulfilled the inclusion criteria.

The following data were collected to obtain information about the inpatient hospitalization:

1. Demographic data such as age at the time of surgery and body mass index (BMI)
2. Previous endometriosis-related surgeries and preoperative pharmacological therapy
3. Surgical access (laparoscopy, laparotomy, conversion), the duration of surgery, the extent of surgical endometriosis lesions excision (complete, incomplete), and intra−/postoperative complications.

Furthermore, the patients were sent a questionnaire by post that was especially developed for the study to obtain data postoperatively. The questionnaire and has not been previously published elsewhere and is provided as Additional File 1.

Questions were asked about the following aspects:

1. Postoperative relief of symptoms as well as postoperative pharmacological therapy, if applicable
2. Development of a recurrence with the need for repeated surgery, including numbers and rASRM stage
3. If the patients had the desire to have children postoperatively: preoperative pregnancies and deliveries as well as postoperative pregnancies, the type of delivery and outcome regarding the child were included.

The statistical analysis was performed with the statistical software R (http://www.cran.r-project.org). After descriptive processing, a correlation analysis was performed, the significance of the correlation was tested and logistic regression was applied. Statistical significance was indicated if the *p*-value was less than 0.05.

## Results

Out of 456 patients who were primarily eligible for inclusion 206 sent back the questionnaires which corresponds to a response rate of 45.2%. These 206 patients were included in the final analysis. A total of 137 (66.5%) had stage III disease during surgery and 69 women (33.5%) had stage IV disease.

### Demographic data

The average BMI was 24.1 (SD ± 5.1; 14–44). The average age at surgery was 37 years (SD ± 7; 17–59), and the majority of patients (*N* = 171; 83.0%) were under the age of 45.

A total of 38.8% of the women (80/260) had already undergone previous surgery for endometriosis. The average number of endometriosis-related previous surgeries was 0.5 (SD ±1.01; 0–9). The stages of previous endometriosis disease could not be analysed separately because they were unknown in most cases.

Preoperative pharmacological therapy was performed in 30.6% (63/206) of patients for 4.4 months on average with a maximum of 10.6 months. The following products were used: GnRH analogues (*N* = 29), gestagen (*N* = 16), combined oral contraceptives (*N* = 13), and unknown substances (*N* = 3). Two patients received substances in combination (a GnRH analogue and a gestagen).

### Surgical data and procedures and intra- and postoperative complications

The overall average duration of surgery was 122 min (SD ± 75.7; 20–424). In 63.1% (*N* = 130) surgery was performed by laparoscopy, with an average duration of surgery of 81 min (SD ± 46.1; 20–424). Laparotomy was performed in 21.8% (*N* = 45) of patients, with an average duration of surgery of 189 min (SD ± 69.1; 65–419). Here, some of the patients also underwent tubal surgical procedures under the surgical microscope so that the laparotomy was planned beforehand. In 31 patients (15%), conversion from laparoscopy to laparotomy took place, and the surgery then lasted 202 min on average (SD ± 48.8; 125–327).

Complete endometriosis lesions excision was achieved in 90.8% of the women (187/206). In 19 women, removal was incomplete (9.2%). Reasons for this incomplete removal included the existing wish to have children, with a high risk of destroying ovaries and tubes or the risk of pronounced intestinal damage.

In a total of 8.3% of the patients (17/206), a surgical intestinal and bladder procedure was performed due to deep infiltrating endometriosis. In three patients (1.5%), endometriosis lesions removal included bladder surgery with partial resection of the bladder and partial resection of the ureter. Fourteen patients (6.8%) underwent intestinal surgery.

A total of 6.8% of the patients (14/206) developed intra- and postoperative complications. In seven of these patients, the complications developed directly during surgical endometriosis lesions removal due to adhesions to the respective anatomical structures including damage to the rectum, bladder, ureter or distal part of the tube, and were treated immediately during surgery. A further seven patients developed general, minor intra- and postoperative complications (renal congestion, voiding dysfunction, wound healing disorders, Quincke’s oedema, hypertensive crisis, iatrogenic injury of the peroneal nerve, cardiovascular instability, subfascial haematoma). Long-term morbidity did not occur.

### Postoperative interventions, decrease in symptoms and the rate of recurrence

A total of 51.5% of the patients (106/206) received postoperative pharmacological therapy for 26.9 months on average (SD ± 33.2). In 94.3%, the therapy consisted only of one single active substance: a GnRH analogue, a gestagen preparation or combined oral hormones/contraceptives. Six of the 106 women received a combination of several of the abovementioned active substances after surgery. A total of 100 women (48.5%) did not receive postoperative pharmacological therapy. The main reason for this was indicated as the active wish to have children directly after surgery.

Complete relief of symptoms was achieved in 76.6% (157/205) of women who answered this question. Partial relief of symptoms was achieved in 16.6% (34/205). Fourteen patients (6.8%) did not show any postoperative improvement. Symptoms were specified as dysmenorrhoea, intermittent bleeding, dyspareunia, hypermenorrhoea, pain during urination and defecation, ovulation pain, pain in the pelvic and sacral region, lower abdominal pain irrespective of the cycle and increased sensitivity to pain in the lower abdomen.

The stage of endometriosis had no effect on the relief of symptoms: 104 patients with stage III (75.9%) and 53 patients with stage IV (76.8%) developed symptom relief.

The rate of recurrence was 21.8% (45/206); therefore, surgery was necessary again over the course of follow-up.

### Effect of stage and surgical access on the rate of recurrence

A total of 22.6% of the patients (31/137) in stage III after surgery and 20.3% (14/69) of patients in stage IV developed recurrence (*p*-value = 0.7).

In patients with disease recurrence, laparoscopy was performed in 68.9% of cases (31/45), laparotomy in 22.2% (10/45) and conversion in 8.9% (4/45). The statistical analysis showed no association between the rate of recurrence and surgical access (*p*-value = 0.4). Additionally, with regard to the issue of a combined association among the rate of recurrence, stage and surgical access, no statistical significance was established.

### Postoperative rate of recurrence depending on the extent of excision

After incomplete resection of endometriosis lesions, the rate of recurrence was 36.8% (7/19); after complete resection, the rate was only 20.3% (38/187). With 0.104, the *p*-value of the regression coefficient was slightly over the threshold of significance.

After receiving postoperative pharmacological therapy, 29 of 106 women suffered a recurrence (27.4%), whereas without subsequent treatment, recurrence occurred in 16% (16/100). This difference is statistically significant (*p*-value = 0.0496).

In the multivariate logistic regression analysis the variables maternal age, stage of endometriosis, surgical access, the extent of excision and postoperative pharmacological therapy are taken into consideration simultaneously. Statistically significant factors that were associated with a higher risk to develop recurrence were a lower maternal age (*p*-value = 0.00474) and the use of postoperative pharmacological therapy (*p*-value = 0.04993).

### Postoperative fertility: wish to have children, pregnancy and birth rate, and neonatal outcome

Following surgical removal of the endometriosis lesions, 120 patients (58.2%) had the desire to have children, and the majority were under 45 years of age (*N* = 117; 97.5%). Seventy-nine women became pregnant at least once as wished.

Approximately 70.4% (57/81) of the women after resection of stage III endometriosis lesions and 56.4% after resection of stage IV endometriosis lesions (22/39 women) became pregnant. Statistical significance was not observed here (*p*-value = 0.131). Overall, the postoperative pregnancy rate was 65.8% (79/120).

Twenty-nine women conceived twice and one patient each three, four and five times, respectively, after surgery. The average number of pregnancies was 1.4 (SD ± 0.7; 1–5).

The delivery rate (all birth modes) for the first pregnancy was 80.5% at the time of the survey. The children were born on average in the 39 + 3/7 week of gestation, with an average weight for singleton pregnancies of 3254 g. There were four twin pregnancies. At the time of the survey, two women were still pregnant in their first pregnancy. The delivery rate for the 2nd postoperative pregnancy was 92.3%.

A total of 76.0% of women who had been pregnant at least once before surgery became pregnant after surgery, while 61.4% of nulligravidae were able to conceive. Statistical significance was not seen here (*p*-value = 0.4556).

### Influence of age, the stage of endometriosis, surgical access and the extent of excision on postoperative fertility

A total of 76.7% of women up to and including the age of 34 (56/73) and 48.9% of women from the age of 35 up wishing to have children (23/47) became pregnant after surgery (*p*-value = 0.0017). This means that the chances of conception after surgery were significantly increased at a younger age up to 34 years.

After laparoscopic endometriosis surgery, 74.3% (52/70) of women became pregnant after surgery, 61.3% (19/31) became pregnant after a laparotomy and 42.1% (8/19) became pregnant after a conversion from laparoscopy to laparotomy. There was a statistically significant association between postoperative pregnancy rate and surgical access (*p*-value = 0.00893).

After incomplete resection, 41.5% (5/12) of women became pregnant, while 68.5% (74/180) of women were able to conceive after complete surgical excision.

With 0.063, the p-value of the regression coefficient between the extent of removal and the postoperative pregnancy rate was slightly over the threshold of significance. Complete removal of the endometriosis lesions may increase the chance of future pregnancy.

In the multivariate logistic regression analysis the variables maternal age, stage of endometriosis, surgical access and the extent of excision are taken into consideration simultaneously. The dominant statistically significant factor that was associated with a higher postoperative pregnancy rate was the age < 35 (*p*-value = 0.00267).

Table 1 summarizes the main results of the study.

**Table 1:** Main results of the study

	Proportions of patients in percent (number)		
History of endometriosis surgery	38.8 (80/206)		
Preoperative medical treatment	30.6 (63/206)		
Preoperative pregnancies	39.8 (82/206)		
Postoperative medical treatment	51.5% (106/206)		
Postoperative relief of symptoms (complete and partial)	93.2% (191/206)		
Postoperative recurrence rate	21.8% (45/206)		
Postoperative pregnancy rate if women had the desire to have children (*N* = 120)	65.8% (79/120)		
Postoperative birth rate in first and second pregnancy with regard to all pregnancies	80.5% (first) 92.3% (second)		
Postoperative total pregnancy rate depending from maternal age	≥34 years: 76.7% (56/73) ≤35 years 48.9% (23/47)		
	**Stage III (number)**	**Stage IV (number)**	
Intraoperative stage of endometriosis (rASRM)	66.5 (137/206)	33.5 (69/206)	
Postoperative recurrence of disease	22.6 (31/137)	20.3 (14/69)	
Postoperative pregnancy rate if women had the desire to have children (*N* = 120)	70.4% (57/81)	56.4% (22/39)	
	**Laparoscopy** **(number)**	**Laparotomy** **(number)**	**Conversion** **(number)**
Duration of surgery in minutes	81	189	202
Proportion of patients in percent (N)	63.1 (130)	21.8 (45)	15 (31)
Postoperative recurrence rate with regard to surgical approach	23.8 (31/130)	22.2 (10/45)	12.9 (4/31)
Postoperative recurrence rate with regard to total number of recurrence (*N* = 45)	68.9 (31/45)	22.2 (10/45)	8.9 (4/45)
Postoperative pregnancy rate if women had the desire to have children (*N* = 120)	74.3% (52/70)	61.3% (19/31)	42.1% (8/19)
	**Proportions of patients in percent (number)**		
Proportion of complete resection of endometriosis in percent (N)	90.8 (187/206)	thereof postoperative recurrence: 20.3% (38/187)	thereof postoperative pregnancy 68.5% (74/180)
Proportion of incomplete resection of endometriosis in percent (N)	9.2 (19/206)	thereof postoperative recurrence: 36.8% (7/19)	thereof postoperative pregnancy: 41.5% (5/12)
Proportion of intestinal and/or urological surgeries in percent (N)	8.3 (17/206)		
Proportion of intra- and postoperative complications in percent (N)	6.8 (14/206)		

## Discussion

In this retrospective single center study presented here, we analysed the rate of recurrence of 206 premenopausal patients who underwent surgery for moderate to severe endometriosis in a period of 11 years and who completed the questionnaire sent to them. Unfortunately, the majority of the 456 patients that were primarily eligible for inclusion could no longer be contacted by post or telephone. Common benign comorbidities did not exclude the patients from the analysis, which, to our mind, represents a real cross section of the normal population.

We also analysed the pregnancy rate for 120 women who wanted to become pregnant after surgery. The majority of these women was under the age of 45 (*N* = 117; 97.5%).

Compared to other publications, this is a high number of patients. Recently, Sun et al. published a study with 59 infertile patients after laparoscopic cystectomy with six to 10 years of follow-up [24].

Our long follow-up period of four to 14 years after the index surgery enabled the registration and evaluation of the long-term efficacy of surgical endometriosis lesions resection with regard to the development of a recurrence and postoperative pregnancy. However, as a limitation of our study, the long follow-up period was problematic to the extent that a number of patients could no longer be reached per mail or telephone or through the attending physicians. After contacting 456 of the patients enrolled in the study, the return rate of the questionnaires was 45.2%. This is, however, in the higher range when compared to other questionnaire-based studies [25–27]. We are aware that in these 11 years there were changes in term of diagnostic tools and detailed choice of postoperative treatment. Though, the principles of pharmacological therapy and as well the qualifications and skills of the operative personnel remained unchanged.

As Haas et al. [28] have shown in their retrospective epidemiological study, endometriosis affects young adolescents as well as perimenopausal women. Our study also included these women, so that overall, a cross section of all affected age groups was achieved. Obviously the fertility capacity decreases considerably over the age of 45 years; therefore, the low number of three women over the age of 45 years who were trying to become pregnant after surgery was to be expected.

Irrespective of the high number of patients analysed in this paper, we are fully aware that the retrospective approach and the questionnaire-based approach of the study limits the value of the results.

### Stage-related surgical procedures and postoperative rate of recurrence

Surgical treatment for endometriosis is technically challenging and is sometimes similar in its complexity to the radical surgeries in gynaecological oncology. During the excision of extensive endometriosis lesions, close attention must be paid to the preferably complete removal of the endometriosis lesions and the preservation of the functional, reproductive structures in the minor pelvis. Therefore, especially in women wanting to have children, patient age, previous pharmacological or surgical therapies and the extent of the disease must therefore be considered [29, 30]. Furthermore, the benefits of excessive surgery before IVF/ICSI are still uncertain and must be weighted carefully against the risks [15].

Complete surgical removal of endometriosis lesions was achieved in 90.8% (187/206) of our study population, which is – with respect to the high stages of disease – a high proportion. The most common reason for incomplete resection was the patient’s existing wish to have children with the relevant high risk of serious destruction of the ovaries and tubes.

With 6.8%, the peri- and postoperative rate of rather minor complications was low despite the partly very extensive disease and is in line with or even lower than the data published in literature [20, 30–32]. In half of these patients, the complications were associated with the immediate operative distance of the endometriosis lesions at the respective anatomical structures. The most common postoperative complication described was renal congestion [20], which developed in 1% of our study population.

The complete or partial relief of symptoms associated with endometriosis was achieved in 93.2% of our patient population, whereby complete remission of symptoms accounted for the largest percentage with 76.6%. This result is high when compared with other published data [33–35]. We were not able to establish any differences in symptom relief regarding the rASRM stage, which also corresponds to the results of Vercellini et al. [36]. Comptour et al. [37] also demonstrated that laparoscopic endometriosis lesions resection irrespective of the rASRM stage has a positive, minimum three-year effect on both symptoms (lower abdominal pain, dyspareunia) and the quality of life.

A recurrence that led to repeated surgery developed in 21.8% of patients in our sturdy population. This value falls into the lower range of data published in the literature, where a recurrence rate of between 18.9 and 57% after 5 years is specified [18–20, 22, 38–40]. It should be kept in mind that in some of our patients, 14 years had passed since surgery.

We were not able to establish a statistically significant association between recurrence rate and disease stage; there is discord in the literature in this regard. While Vercellini et al. [36] were not able to demonstrate an association between stage and risk of recurrence and attribute a rather low predictive power to the rASRM classification, Busacca et al. [41, 42] showed that both the rASRM stage as well as previous surgeries constituted influencing factors for recurrence. Tobiume et al. published data from 352 patients who underwent surgery of endometrioma and determined that only the rASRM score was correlated with a recurrence of 28.7% after 5 years [40].

Possible reasons for this discrepancy can be the difference in the technical performance of the surgery or the expertise of the surgeon, the definition of the measured endpoint (symptom relief or repeated surgery) as well as the length of the follow-up period and the patient population [19]. The studies of Busacca et al. [41, 42] focused on patients with stage III and IV endometriosis but defined the development of recurrence by means of postoperative sonographic findings and questionnaires without histological and/or laparoscopic confirmation. Vercellini et al. [36], on the other hand, examined patients of all rASRM stages and defined the development of recurrence by means of sonographic findings and a new surgical intervention. The findings of our study essentially correspond with the results of Vercellini et al. [36]. Our study only concentrated only on patients with moderate and severe endometriosis where the development of recurrence was defined by the need for new surgery with histological confirmation.

Even if the initial stage of endometriosis had no effect on recurrence in our study, recurrence developed more often in those with incomplete resection with 36.8% than in those with complete resection (20.3%). However, this difference was not significant in our study, neither in univariate nor in multivariate analysis. These observations correspond with other publications [19, 39, 43].

Hormonal follow-up treatments are indicated after endometriosis surgery since they verifiably decrease the recurrence rates [43–46]. Thirty-five women in our population (33.0%) received postoperative therapy with GnRH agonists, often as pre- and postoperative ‘sandwich therapy’, to ensure treatment success and optimize the likelihood of becoming pregnant in the long term.

A total of 100 women (48.5%) in our population did not receive postoperative pharmacological therapy. In 53%, the reason for this was indicated as the active wish to have children directly after surgery. In 22.8% (47/206), adequate postoperative pharmacological therapy was not administered or taken, which should be improved.

Surprisingly, the use of postoperative pharmacological therapy was associated with a higher risk to develop recurrence in multivariate logistic regression analysis. This may be explained by the fact that a high percentage of patients became pregnant after surgery, some several times, which may lower the risk of recurrence during the study time. Secondly, the duration and drug of pharmacological therapy was heterogeneous. The current gold standard for surgical endometriosis lesions resection is the laparoscopy. This was performed in a total of 63.1% of interventions in our patient population. In our study, a laparotomy was performed in 21.8% of patients. Mostly, this was intended beforehand since fertility-preserving and restorative interventions on the tubes and uterus are performed depending on the equipment of the particular hospital, as the use of a surgical microscope and microsurgical instruments is necessary.

Surgical access, on the other hand, had no effect on the rate of recurrence in our patient population. This observation corresponds to international data [47, 48].

### Stage-dependent postoperative pregnancy rate

The postoperative pregnancy rate in our study was 65.8% (79/120) if the women wanted to have children. The average number of pregnancies was 1.4 and therefore higher than the postoperative pregnancy rates described in the literature, which are between 32.4 and 65.5% after the excision of a stage III and IV endometriosis lesions [24, 39, 41, 49, 50]. The birth rate was 80.5%, and a previous pregnancy improved the chance of becoming pregnant again after surgery (76.0% versus 61.4%).

As expected, we were able to determine a statistically significant correlation between the onset of pregnancy and maternal age (*p* < 0.0017) in univariate analysis. Furthermore, a lower age < 35 was the dominant statistically significant factor in multivariate analysis which was associated with a higher postoperative pregnancy rate (*p* < 0.003).

A total of 76.7% of women up to the age of 34 years became pregnant, while only 48.9% of women from the age of 35 up were able to conceive. The fertility capacity decreases as a women age, and moreover, with the resection of ovarian endometrioma, there is a risk of reduction in the ovarian reserve and thus less likelihood of conceiving [50].

In most studies investigating the effect of surgical therapy of endometriosis with regard to several outcome parameters such as symptom relief, recurrence rate and pregnancy rate, the surgery was performed as laparoscopy [25, 33, 36, 39, 51]. A prospective study compared the effect of the resection of colorectal endometriosis lesions with laparoscopy and laparotomy on fertility and showed that the rate of spontaneous postoperative pregnancies was distinctly higher in the group with laparoscopic access [52]. Daraï et al. also showed that laparoscopic endometriosis lesions resection is associated with higher postoperative pregnancy rates than resection via laparotomy, and has similar positive effects on symptoms and the quality of life [53].

Our results substantiate these findings with regard to postoperative pregnancy rates after laparoscopic surgery, which were statistically higher (74.3%) than after laparotomy (61.3%) as well as after conversion surgery (42.1%). The advantage of laparoscopy is likely to be found in the low formation of adhesions, since the tissue damage is lower overall [54]. In our collective patients, we had a high percentage of women with severe tubal damage who were treated by laparotomy to restore fertility, using surgical microscopy and microsurgical instruments. Possibly, the lower rate of postoperative pregnancy after laparotomy was caused by these unfavourable conditions of the fallopian tubes.

After complete resection of endometriosis lesions, the pregnancy rate was higher (68.5%) than after incomplete excision at 41.7%. However, the correlation was not statistically significant in the multivariate logistic regression analysis. The retrospective cohort study of Soriano et al. [51] was able to demonstrate that radical surgical removal that also included a procedure for intestinal and bladder surgery does not have to involve a decrease in fertility. Surgical therapy is able to restore the pelvic anatomy that is changed by endometriosis [10]. However, it is essential to strike a balance between complete resection and the preservation of the reproductive organs and their function. In addition to the risk of diminishing the ovarian reserve, attention must be paid to the formation of new or rather further adhesions [55].

A new and future possibility to preserve fertility can be oocyte vitrification for patients not only in oncological situations but also in situations such as severe endometriosis that may induce the risk of premature ovarian failure [56–58].

## Conclusion

We found a high percentage of 65.8% of women who became pregnant, sometimes several times, after the excision of moderate or severe endometriosis lesions, a low intra- and postoperative rate of complications of 6.8%, a high rate of complete or partial postoperative reduction in symptoms of 93.2% and a low rate of recurrence of 21.8% compared to international literature, which are very encouraging values. Some of our patients had undergone several previous surgeries, were already older with regard to fertility with an average age of 37 years and were surveyed sometimes after a very long postoperative interval.

Our results show that patients with moderate or severe endometriosis, individually tailored therapy concepts to preserve fertility and decrease the risk of recurrence can be promising.

## Supplementary information

**Additional file 1.** Patient’s questionnaire.

## Data Availability

The dataset that support the findings of this article belong to the Hannover Medical School. The survey data are not publicly available but can be obtained from the corresponding author upon reasonable request. The qualitative data cannot be made available in order to protect the confidentiality of study participants.

## Abbreviations

rASRM: Revised American Society for Reproductive Medicine
AMH: Anti Mullerian Hormone
GnRH: Gonadotropin Releasing Hormone
IVF: In-vitro fertilization
ICSI: Intracytoplasmatic sperm injection
ESHRE: European Society of Human Reproduction and Embryology
