# Supplementary material for: Reproductive capacity and recurrence of disease after surgery for moderate and severe endometriosis – a retrospective single center analysis
Source: BMC Womens Health. 2020 Jul 13;20:144. doi: 10.1186/s12905-020-01016-3 (PMC7358195; doi:10.1186/s12905-020-01016-3)
Supplement: Supplementary file 1 — Additional file 1. Patient’s questionnaire. [file 12905_2020_1016_MOESM1_ESM.docx]

**Questionnaire for patients after**

**endometriosis surgery in the Department of Obstetrics and Gynaecology, Hannover Medical School**

Last name, First name: ___________________________________________

Surgery date/year (if known):__________________________

**Questions concerning the condition endometriosis:**

**1)** Could your symptoms be alleviated/improved through the surgery performed at Hanover Medical School?

Yes 🞎 No 🞎 partially 🞎:__________________________

**2) After** the surgery, did you receive postoperative medical therapy (e.g. GnRH analogues ["menopause injections"], progestogen-only pill, normal pill or similar)?

Yes 🞎 No 🞎

If yes: which medication and for how long?

Medication: _______________________________, Duration: ________________

**3)**  **After** the endometriosis surgery, did you take advantage of a rehabilitation program or health spa?

Yes 🞎 No 🞎 I had no idea that this is possible 🞎

**4) After** the surgery performed here, did you have to undergo surgery ***again*** due to endometriosis?

Yes 🞎, namely _________ times No 🞎 *🡪 in this case please go to Question 5*

**4a)** If you had to undergo surgery again: Was the surgery performed by endoscopy (laparoscopy) or abdominal section (laparotomy)?

If 1^st^ surgery: endoscopy (laparoscopy) 🞎 Abdominal section (laparotomy) 🞎

If 2^nd^ surgery: endoscopy (laparoscopy) 🞎 Abdominal section (laparotomy) 🞎

**5)** If you underwent surgery again: do you know at what stage (I-IV) your condition was at the time of the repeat surgery?

Stage I 🞎, Stage II 🞎, Stage III 🞎, Stage IV 🞎, Stage unknown 🞎

**Questions concerning the desire to have children:**

**6)** How often were you pregnant **before** the endometriosis surgery? _______ times

**7)** How many children did you give birth to **prior to** the surgery? ________ child/ren

**8)**  Did you want to have children **after** the surgery performed here?

Yes 🞎 No 🞎 *🡪 if no: end of questionnaire, thank you*

**If yes:**

**8a)** Did you get pregnant **after** the surgery?

**Yes 🞎** namely ____ times **No 🞎** *🡪 if no: end of questionnaire, thank you*

**9)** If you got pregnant one or several times **after** the surgery, we ask you to give us some information about the course of the pregnancy and birth:

**1. Pregnancy after the surgery**

The pregnancy ended in the _____^th^ WOP *[week of pregnancy]* (see pregnancy record/birth report)

Child's **birthweight**: _________ grams

**Type of delivery:** 🞎 Normal/spontaneous delivery

🞎 Vacuum extraction ("VE")

🞎 Forceps delivery

🞎 Caesarean section

**2. Pregnancy after the surgery**

The pregnancy ended in the _____^th^ WOP [*week of pregnancy]* (see pregnancy record/birth report)

Child's **birthweight**: _________ grams

**Type of delivery:** 🞎 Normal/spontaneous delivery

🞎 Vacuum extraction ("VE")

🞎 Forceps delivery

🞎 Caesarean section

**Thank you for your help!**

Please return this questionnaire to PD Dr. Schippert in the enclosed, prepaid envelope.

Is there anything else that you would like to comment on? The rest of the page or the reverse side is available for this.
